# Supplementary material for: Mind the gap: Distributed practice enhances performance in a MOBA game
Source: PLoS One. 2022 Oct 14;17(10):e0275843. doi: 10.1371/journal.pone.0275843 (PMC9565695; doi:10.1371/journal.pone.0275843)
Supplement: S1 Appendix — (DOCX) [file pone.0275843.s001.docx]

**Time Series K-Means**

Given its widespread use, the first technique we adopted for estimating the presence of different spacing profiles in our data-set relied on the K-Means algorithm (Macqueen, 1967). More precisely, we leveraged a variation of K-Means suitable for application with time-series data (Temporal K-Means), as provided by the python library tslearn (Tavenard et al., 2020).

The major difference between Temporal and conventional K-Means lies in the use of barycenters instead of centroids. If centroids are "prototypical" points in the high dimensional space provided by the data, barycenters can instead be thought as "prototypical" time series acting as a reference from which the euclidean distances to all the other time series are computed.

In order to select the number of barycenters *b* we generated an elbow plot running the algorithm for a range of 2 to 10 *b* with 1 random initialization for a maximum of 300 iterations (S4 Fig). Following the methodology proposed by Satopa et al. (2011), the optimal *b* = 6 was found by individuating the point of maximum curvature in the aforementioned elbow plot. In order to derive interpretable profiles from the individuated cluster, we averaged the time series of between-match time gaps (along with GPM and KDA) over the labels provided by the Mini Batch K-Means. Results of this clustering analysis can be seen in S5 Fig and S6 Fig.

**Density-Based Clustering**

Given the structure observed in Figure 4, we hypothesized that a density-based cluster analysis would have been appropriated for individuating different types of spacing profiles.

As a first step in this analysis we employed UMAP as a feature extractor and reduced the original dataset to a matrix of size *N* x 20. This was done in order to facilitate the convergence of the density based clustering by simultaneously reducing the dimensionality of the data while maintaining the type of topological organization presented in Figure 4. The parameters of the UMAP algorithm for this step were kept equal to those in the visualization stage, except for the number of target dimensions which was changed from 2 to 20.

In order to perform density-based clustering we adopted the Hierarchical Density-Based Spatial Clustering of Applications with Noise (HDBSCAN) algorithm (Campello et al., 2013) as provided by the homonymous python library (McInnes et al., 2018). Some of the advantages of HDBSCAN are that the algorithm does not pose strong constraints on the shape of the clusters (e.g., sphericity in K-Means), it is highly scalable and does not require a-priori specification of the number of clusters.

The algorithm individuates clusters as areas of high density surrounded by low density noise, meaning that not all the the entries in a dataset will be assigned to a specific cluster. HDBSCAN first estimates, though a k-nearest neighbour strategy, densities in different areas of the data space. The k-nearest neighbour step is controlled by a "minimum samples" hyper-parameter which influences the ammount of points considered as noise. Subsequently, the algorithm proceeds to extract clusters through a hierarchical procedure: it will progressively merge points together based on how close they are in the data space (areas where many points are close to each other can be considered to be areas of high density). This process is done by performing the merging procedure at different distance thresholds, generating a complex hierarchy of progressively large clusters.

This hierarchical structure is then condensed accordingly to a "minimum cluster size" hyper-parameter (i.e., the minimum number of points that a cluster needs to possess) and a number of flat clusters are extracted. The clusters selected are those that, according to a hyper-parameter *λ*, are more persistent (i.e., they do not frequently split) in the hierarchy.

We ran HDBSCAN with a minimum samples of 200 and a minimum cluster size of 4000, all the other hyperparameters have been left at the default value provided by the library. In order to derive interpretable profiles from the individuated cluster, we averaged the time series of between-match time gaps (along with GPM and KDA) over the labels provided by the algorithm. Results of this clustering analysis can be seen in S6 Fig and S7 Fig.

**References**

Campello RJGB, Moulavi D, Sander J. Density-Based Clustering Based on Density Estimates. In: Pei J, Tseng VS, Cao L, Motoda H, Xu G, editors. PAKDD (2). vol. 7819 of Lecture Notes in Computer Science. Springer; 2013. p. 160–172.

Available from: http://dblp.uni-trier.de/db/conf/pakdd/pakdd2013-2.html#CampelloMS13.

MacQueen J. Some methods for classification and analysis of multivariate observations. In: of the Fifth Berkeley Symposium on Mathematical Statistics and Probability, Volume 1: Statistics. University of California Press; 1967. p. 281–297.

Available from: https://projecteuclid.org/euclid.bsmsp/1200512992.

Mcinnes L, Healy J, Saul N, Großberger L. UMAP: Uniform Manifold Approximation and Projection. of Open Source Software. 2018;3(29):861. doi:10.21105/joss.00861.

Satopaa V, Albrecht JR, Irwin DE, Raghavan B. Finding a ”Kneedle” in a Haystack: Detecting Knee Points in System Behavior. 2011 31st International Conference on Distributed Computing Systems Workshops. 2011; p. 166–171.

Tavenard R, Faouzi J, Vandewiele G, Divo F, Androz G, Holtz C, et al. Tslearn, Machine Learning Toolkit for Time Series Data. Journal of Machine Learning Research. 2020;21(118):1–6.
