# Supplementary figures and images for: Mind the gap: Distributed practice enhances performance in a MOBA game
Source: PLoS One. 2022 Oct 14;17(10):e0275843. doi: 10.1371/journal.pone.0275843 (PMC9565695; doi:10.1371/journal.pone.0275843)

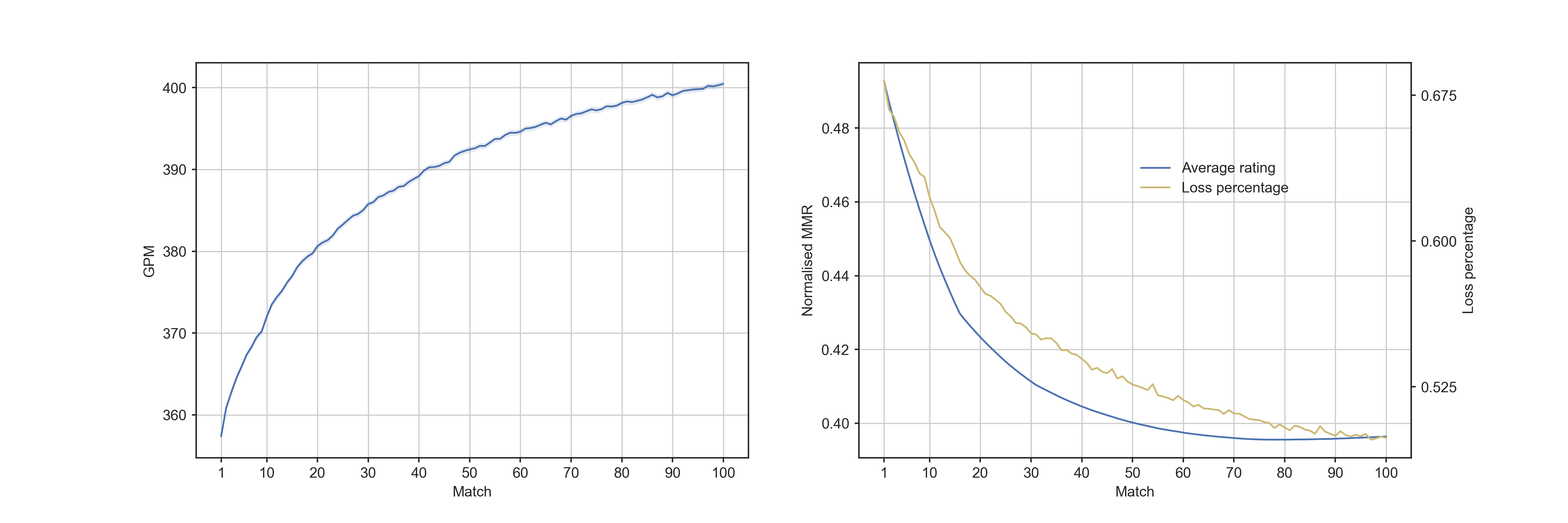

Supplement: S1 Fig — (PNG) [file pone.0275843.s002.png]

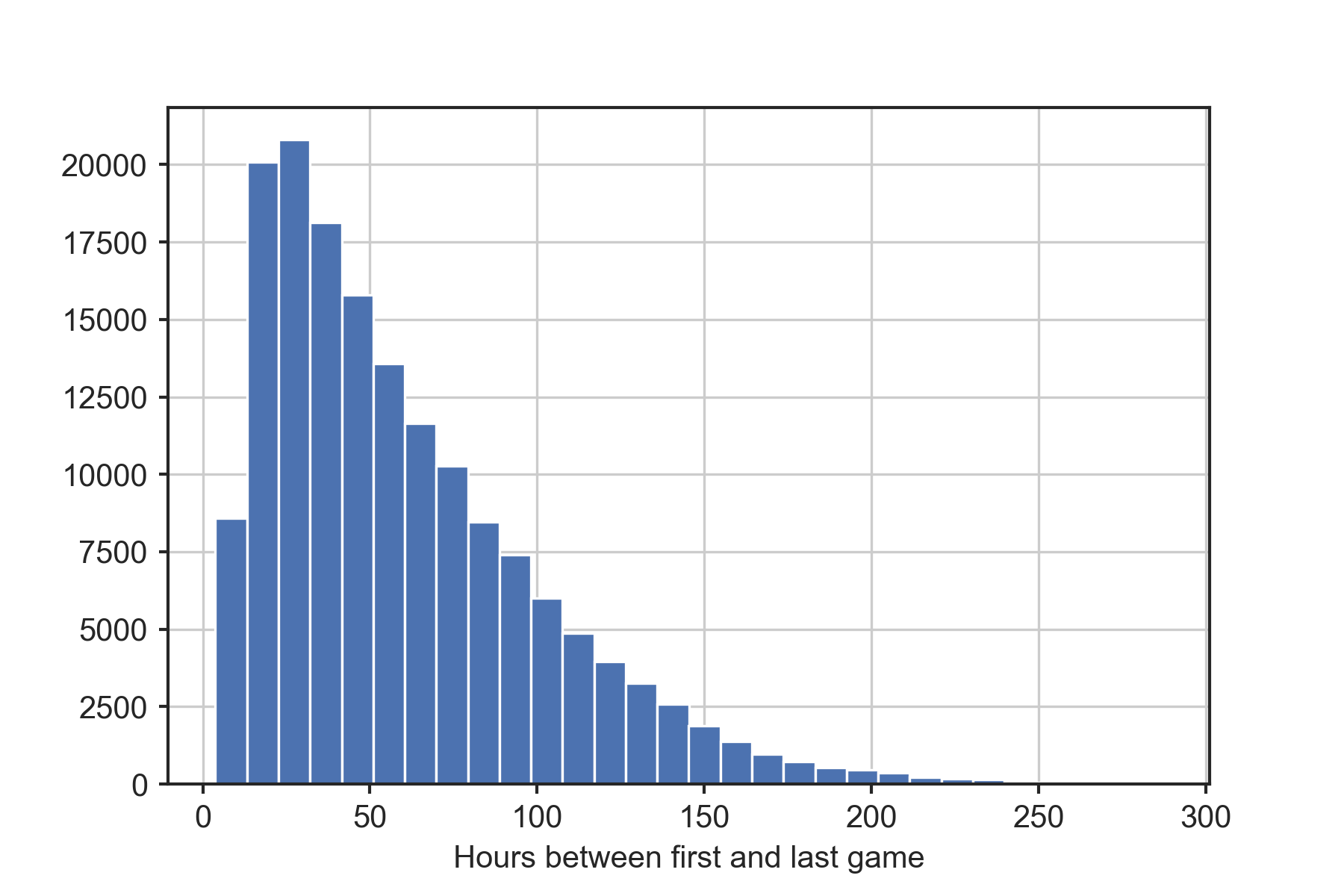

Supplement: S2 Fig — (PNG) [file pone.0275843.s003.png]

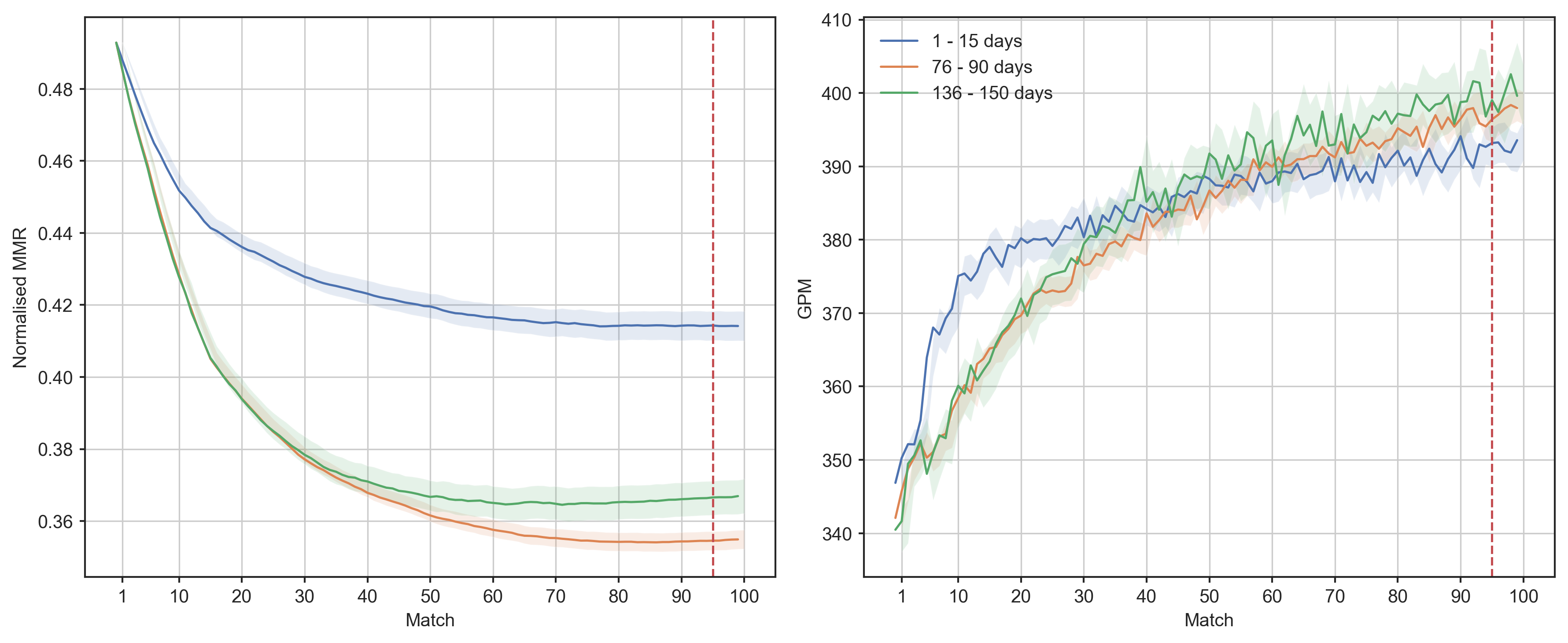

Supplement: S3 Fig — Players in this figure are a subsample who initiate at a similar range of GPM and KDA respectively (approximately surrounding the original sample median). Shaded regions indicate 95% confidence intervals. (PNG) [file pone.0275843.s004.png]

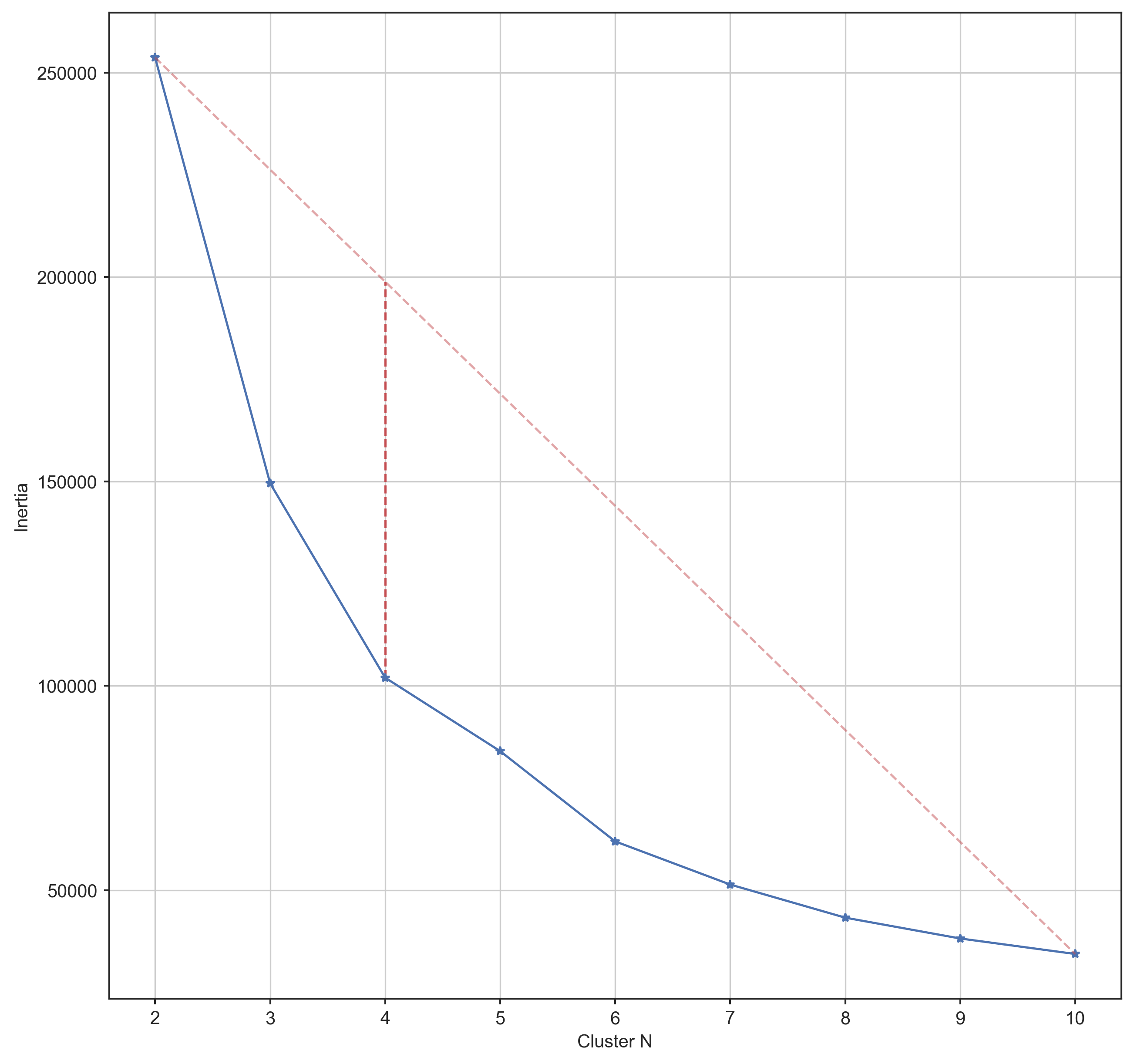

Supplement: S4 Fig — The dotted vertical red line indicates the point of maximum curvature and thus the selected number of optimal k clusters for our K-means clustering of gameplay schedules. (PNG) [file pone.0275843.s005.png]

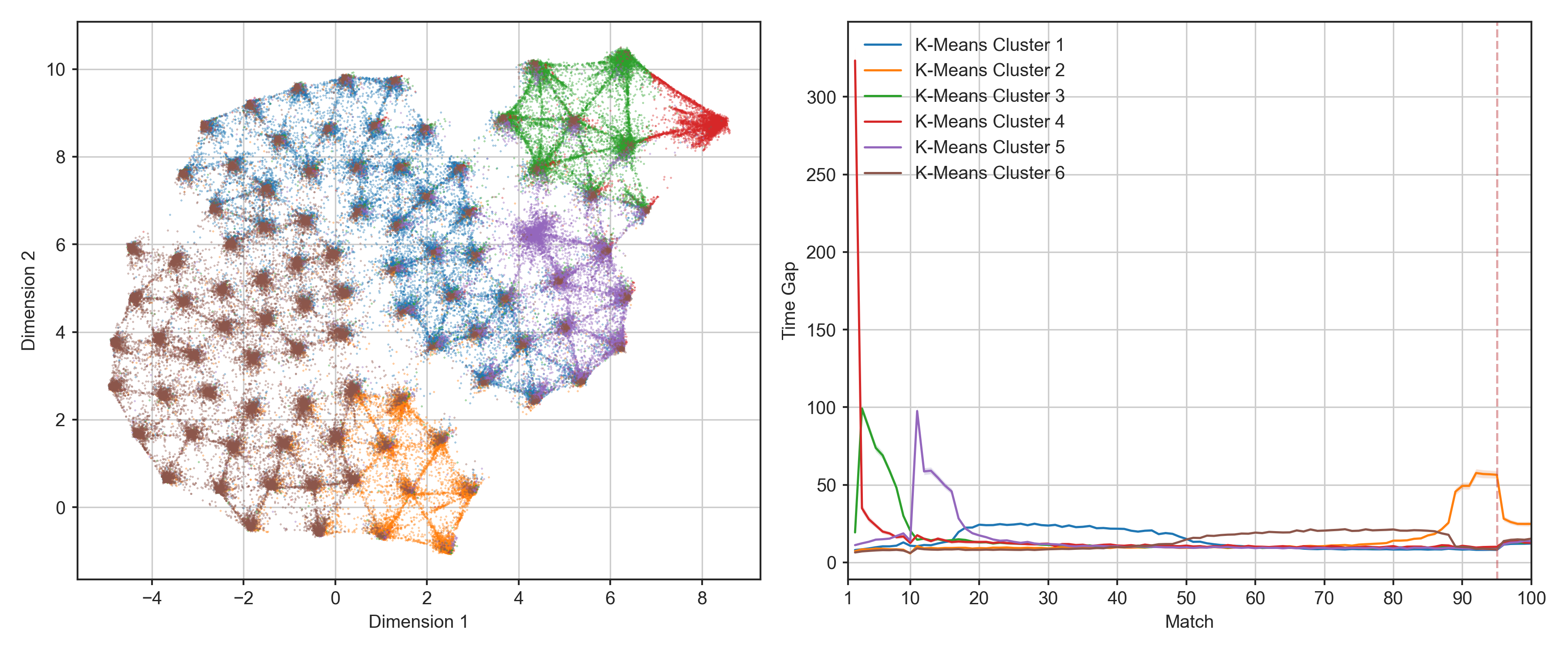

Supplement: S5 Fig — The y and x axes represent the two dimensions individuated by UMAP. The associated values should be interpreted as coordinates on a plane rather than indicators of the magnitude of the two components. Each dot represents the history of inter-match gaps in hours for a single player while distance between dots indicate the degree of similarity between different patterns of spacing. The right panel shows the average evolution of inter-match gap in hours for each K-means Cluster. The y axis indicates the time in hours elapsed since the previous match while the x axis indicates the order of the match. The solid line indicates the mean value while shaded regions show 95% confidence intervals of the mean. The dotted red line separates the observation period (i.e., the first 95 matches) from the evaluation period (i.e., the last 5 matches). (PNG) [file pone.0275843.s006.png]

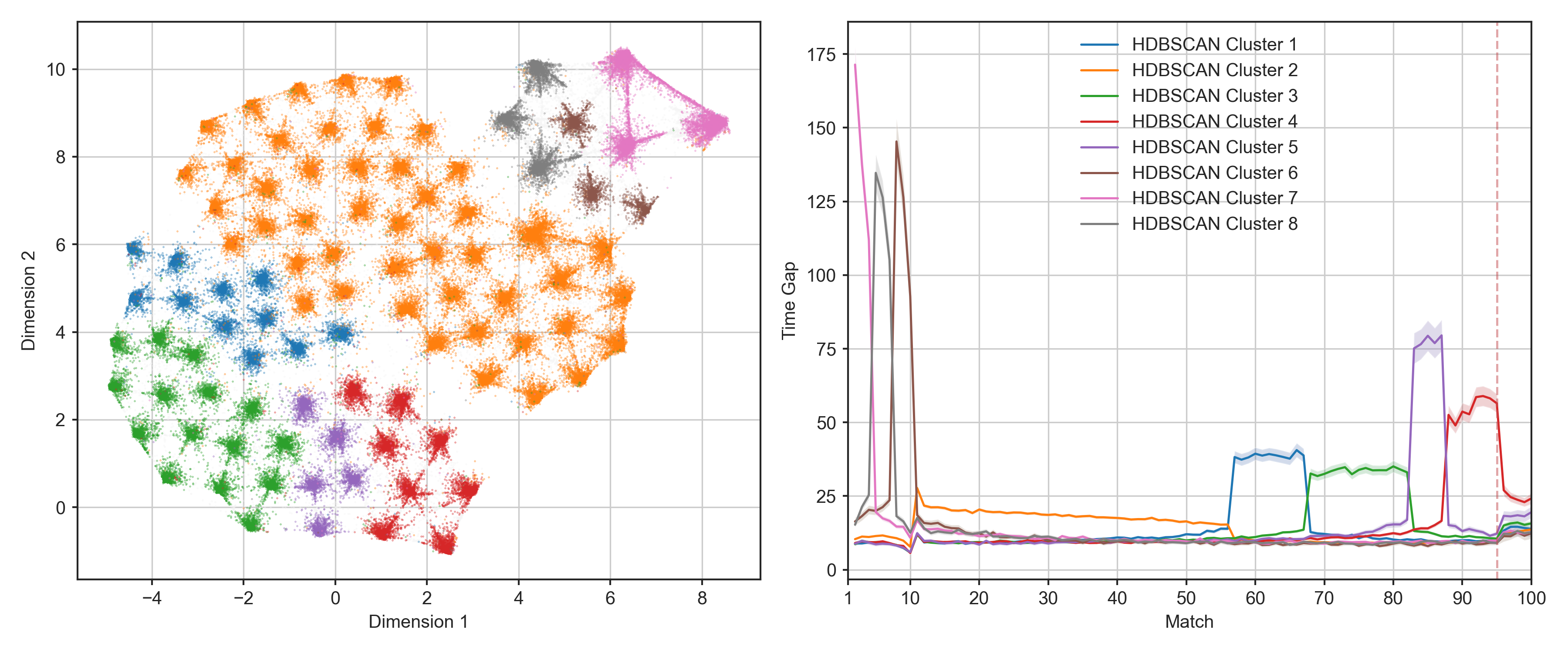

Supplement: S6 Fig — The y and x axes represent the two dimensions individuated by UMAP. The associated values should be interpreted as coordinates on a plane rather than indicators of the magnitude of the two components. Each dot represents the history of inter-match gaps in hours for a single player while distance between dots indicate the degree of similarity between different patterns of spacing. The right panel shows the average evolution of inter-match gap in hours for each density-based cluster. The y axis indicates the time in hours elapsed since the previous match while the x axis indicates the order of the match. The solid line indicates the mean value while shaded regions show 95% confidence intervals of the mean. The dotted red line separates the observation period (i.e., the first 95 matches) from the evaluation period (i.e., the last 5 matches). (PNG) [file pone.0275843.s007.png]

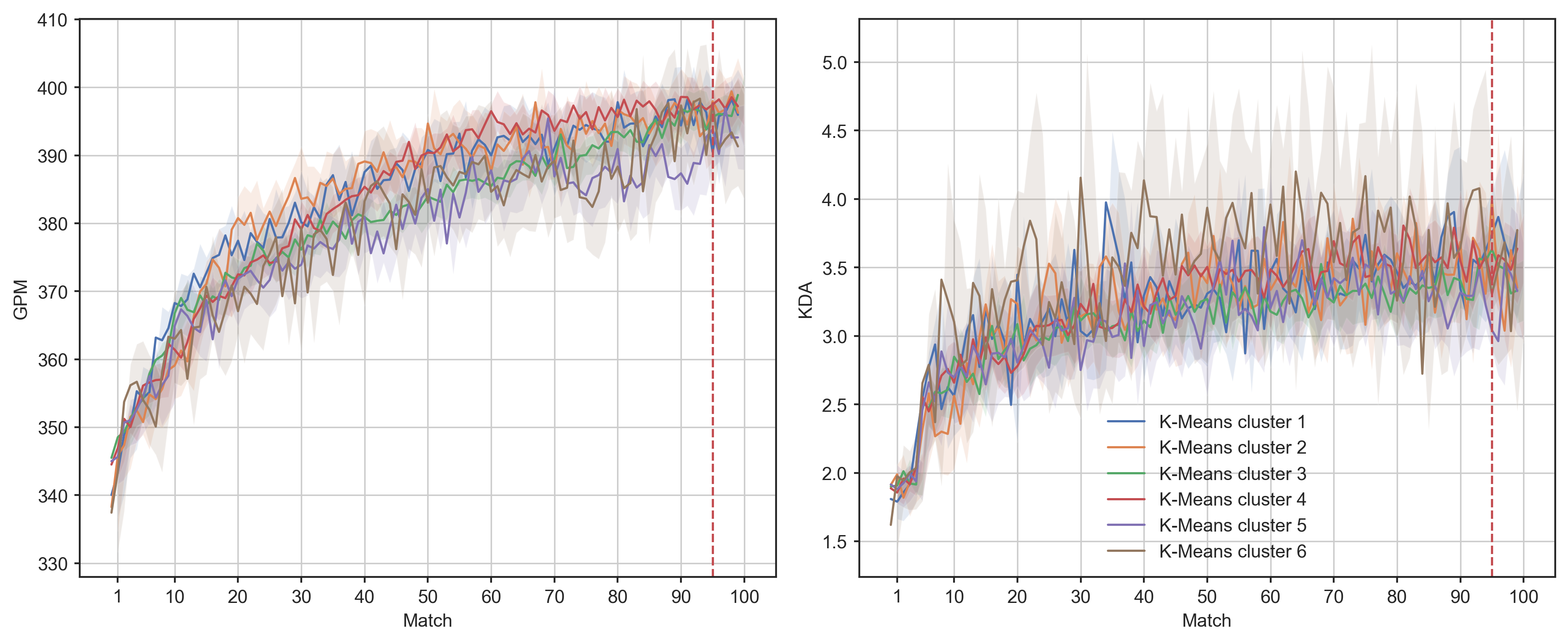

Supplement: S7 Fig — Shaded regions indicate 95% confidence intervals. Players in this figure are a subsample who initiate at a similar range of GPM (approximately surrounding the median of the original sample). (PNG) [file pone.0275843.s008.png]

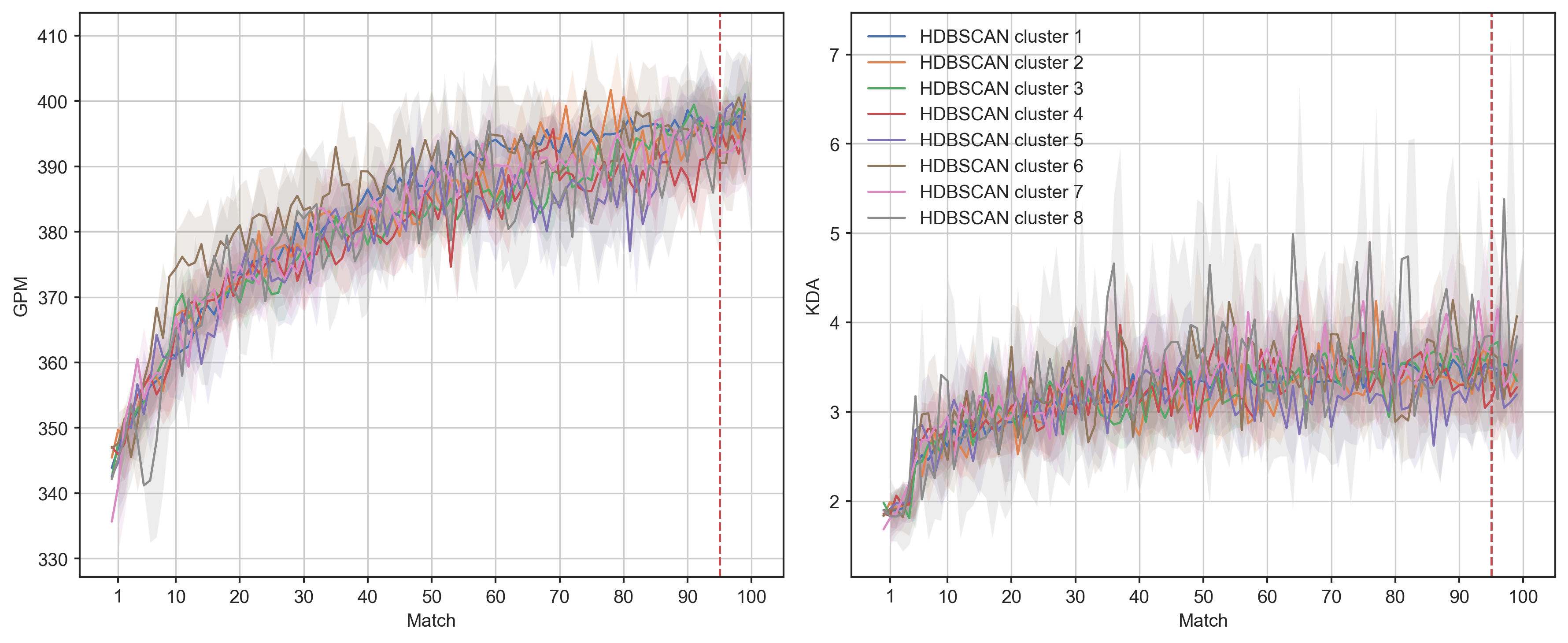

Supplement: S8 Fig — Players in this figure are a subsample who initiate at a similar range of GPM and KDA respectively (approximately surrounding the median of the original sample). Shaded regions indicate 95% confidence intervals. (PNG) [file pone.0275843.s009.png]

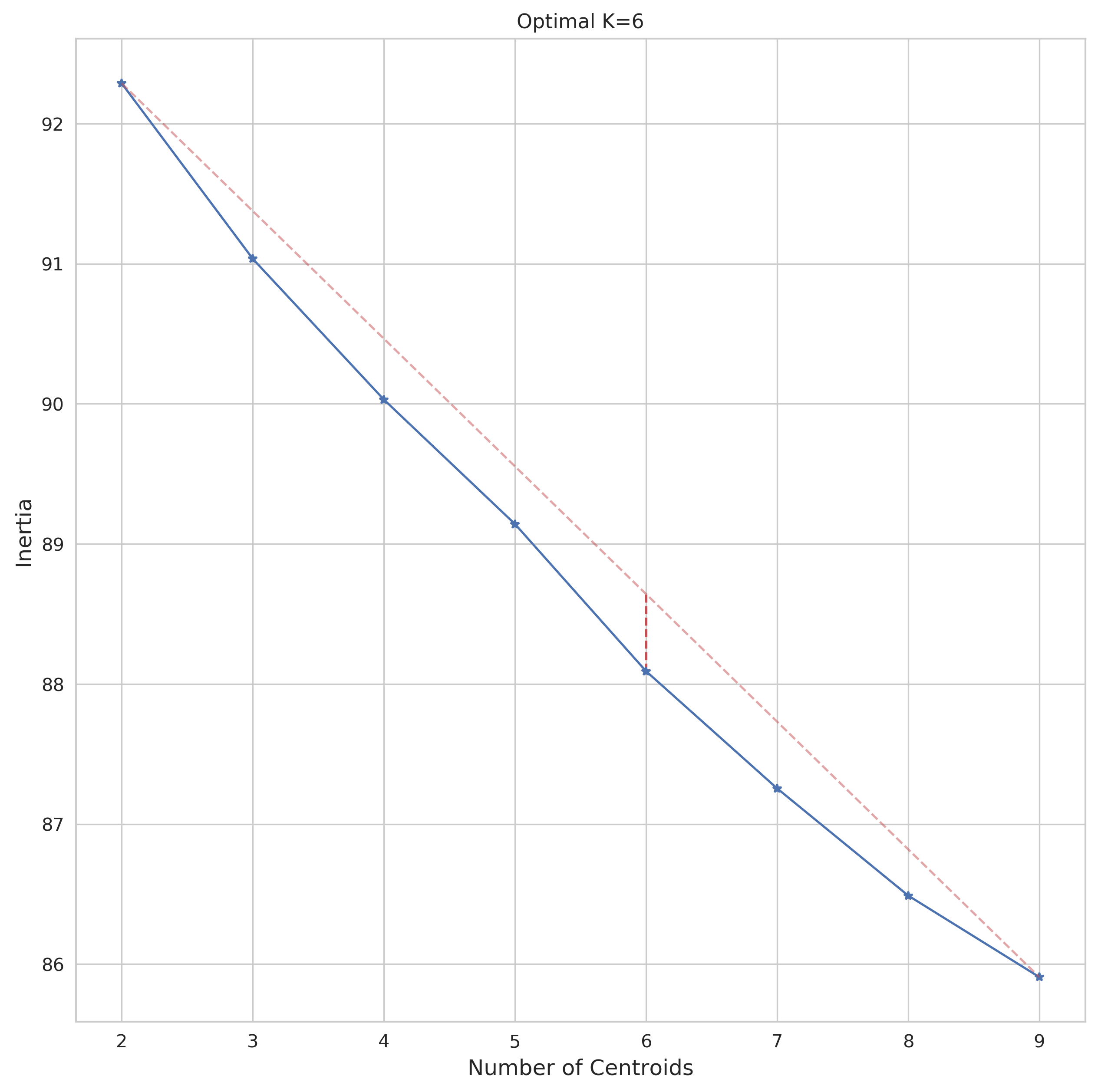

Supplement: S9 Fig — The dotted vertical red line indicates the point of maximum curvature and thus the selected number of optimal k clusters for our K-means clustering of gameplay schedules. (PNG) [file pone.0275843.s010.png]
