# Supplementary material for: Mind the gap: Distributed practice enhances performance in a MOBA game
Source: PLoS One. 2022 Oct 14;17(10):e0275843. doi: 10.1371/journal.pone.0275843 (PMC9565695; doi:10.1371/journal.pone.0275843)
Supplement: Attachment — Submitted filename: PONE-D-22-03288_Rebuttal_Letter.pdf [file pone.0275843.s011.pdf]

PONE-D-22-03288 Revision

Ozan Vardal
Department of Computer Science, University of York

17th May 2022

Dr Musa

Attached is our revision of “Mind the gap: Distributed practice enhances performance in a MOBA game” [PONE-D-22-03288]. We thank the reviewers for their substantive feedback and helpful comments. We have made a number of changes to the submission including further analysis and amendments to figures and text, which we believe reflect the concerns of you and your reviewers. We describe these changes below, commencing with a joint area of concern raised by both reviewers, followed by a point by point discussion of issues raised by individual reviewers.

As before, all analysis code, including the present changes, can be viewed at the following repository: https://github.com/ozvar/lol_practice_distribution. We look forward to further discussion of our paper.

Kind regards,

Ozan Vardal, Valerio Bonometti, Anders Drachen, Alex Wade, Tom Stafford

RE: GPM/KDA will vary by position. An analysis of performance should therefore account for position played.

We thank both reviewers for noting that our analysis of performance could benefit from accounting for the position of the champion that was played. Notably, R1 raises an interesting point that the trajectory of performance exhibited by our spacing group (i.e., depressed initial gains followed by superior final performance) could possibly be explained by players in this group starting their games in the support role, before transitioning to core roles later on.

In order to test this hypothesis we visualised the trajectory of the proportion of matches played in the support role at each *n*th match (see figure below), for each of our spacing groups and for each of our subsamples matched on initial performance (left panel GPM-matched, right panel KDA-matched). Contrary to this hypothesis, we note that players in the group that spaced their first and 95th match the most have the lowest proportion of support games played, but this proportion is consistently low. Thus, our result does not appear to be explained by players transitioning from low earning support roles to high earning core roles. Nevertheless we agree that our analysis is strengthened by devoting additional attention to this aspect of the dataset.

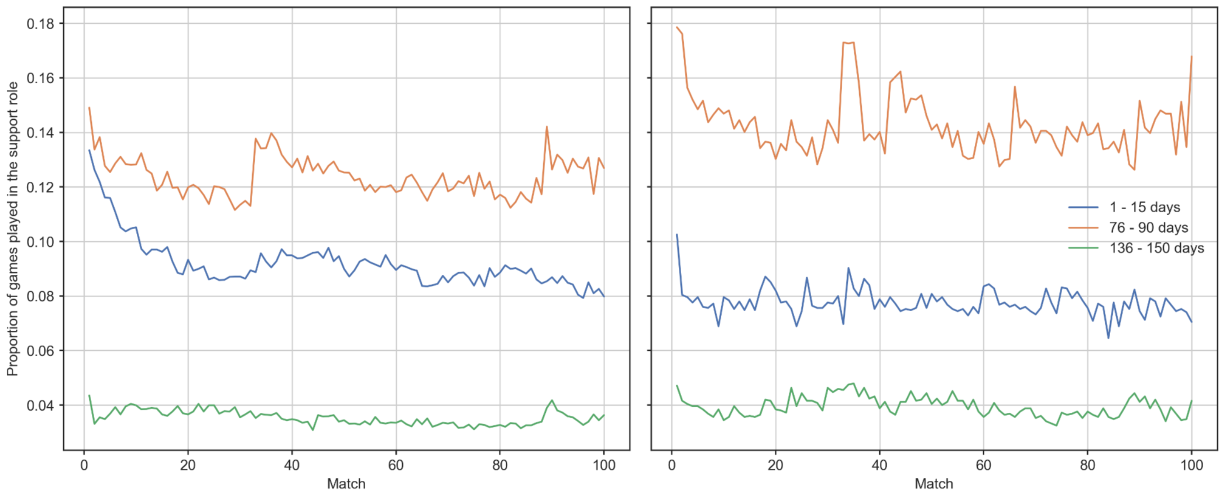

RE: Language edits

Following suggestions by both reviewers and a general proofread, we have corrected several typos including the one suggested by R1 (line 332 in the original manuscript).

Reviewer 1

RE: The final advice for players 4.1 (line 616) is not really useful. In paragraph 1 the authors themselves give both practical and reasons of preference to ignore it.

We have addressed this concern by cutting the mentioned paragraph from the discussion and also the related clause from the abstract. Instead of concluding with policy advice for players, we have ended with a more traditional conclusion section summarising our key findings as well as our recommendations for follow-up research:

Research on motor learning has demonstrated that taking breaks between practice sessions, as opposed to massing them in relatively short time windows, benefits ultimate performance [24,25]. By analysing an observational, longitudinal data set describing player performance in a massive, commercially successful video game, we showed that the distributed practice effect is relevant in an ecologically valid context comprising stakeholders with a vested interested in improving their skills. Although data sets such as ours afford strong statistical power and the ability to filter through observations that meet desired experimental conditions, they are also complicated by noise and potential confounds. As a solution, we propose that researchers seeking to use telemetry data adopt a hybrid approach, collecting demographic information on players before tracking their play records through game APIs. In doing so, interested researchers may control for variables related to initial performance, such as age or cognitive ability [70], and account for sources of data pollution such as players generating data on multiple accounts.

Reviewer 2

RE: Our aim of "generalising laboratory work to an ecologically valid motor skill environment" was not satisfied due to the conflation of practice distribution with frequency

We thank R2 for raising an interesting discussion point regarding the generalisability of our findings. We concede that our work is not a direct translation of past laboratory experiments due to the way we operationalised practice distribution. We add that there *is* some generalisation, as time between individual trials and time between first and last trial is typically correlated.

To clarify this discrepancy, we have amended the initial declaration of aims in our introduction section to the following:

In the current study we extended this line of enquiry to a popular commercial action game, with the aim of generalising work on distributed practice that has been conducted using artificial tasks created by researchers, to a non-artificial, ecologically valid environment with which researchers have not interfered.

We have additionally added the following paragraph in our discussion section to make this limitation clear:

In our case, the absence of experimental control restricted our ability to compare groups of players with homogenous time gaps between each of their play sessions, as has been done in laboratory studies of distributed practice [23]. Our solution, similar to other studies that have used game telemetry [31, 32, 36] was to use a proxy for intersession time interval, namely the time gap between the first and last match. Although time between first and last match is likely related to time between individual trials, we acknowledge that use of this alternative operationalisation limits our ability to generalise from laboratory work to a non-artificial environment.

RE: Introducing additional (SI) results on MMR in the discussion is confusing

We note R2's concern regarding our handling of MMR-related results in the discussion section. To reiterate, our issue with this metric is that the way it is calculated is unknown to anyone besides Riot Games. Although we are aware it is somewhat dependent on match outcome, match outcome itself is dependent on many factors including the behaviour of teammates and opponents. We believe this makes the metric less fit for our purposes than GPM and KDA, which relate more clearly to the performance of the individual at each match.

In light of these reflections we have decided to abridge the paragraph on MMR in the discussion section. To eliminate any related confusion, we have prefaced both the plot (S3 Fig)

introduced in the discussion section as well as our rationale behind discontinuing the analysis of MMR earlier on in the results section as suggested.

RE: Our sample exclusively contains ranked matches. How might players playing unranked matches between ranked ones affect the results?

This is an important point which we acknowledge has not been adequately addressed in our manuscript. We were indeed agnostic to any experience that players may have acquired in unranked game modes that were engaged with between matched that were recorded in our data set. Relatedly, it might be possible that players who spaced their games the most may have been playing unranked matches in between the ranked matches that we observed.

We believe that our results do not support this hypothesis, as we would then have expected the players that spaced their matches the most to have a more accelerated learning curve than what we observed (Figure 2), reflecting the additional practice hours that they accumulated. Instead we observed that players who spaced their matches the most started with a comparatively depressed learning curve. We have included several sentences in the discussion section to make note of this possibility.

RE: Missing information on sample sizes in text

We thank R2 for bringing the absence of vital analysis information to our attention. Firstly, we have added a paragraph at the end of 2.3 clearly explaining the data preprocessing steps that led to us focusing our analysis on a third of the original player sample. Secondly, we have amended the text describing our statistical analysis of KDA so that it mentions the size of that particular subsample. Finally, while checking that each portion of the analysis contains clear descriptions of sample sizes, we noticed that our regression analysis does, and in fact was erroneously conducted on the entire sample as opposed to our subsamples matched on initial performance. We have corrected this portion of the analysis together with all in-text descriptions of results, ensuring that sample sizes are mentioned in all cases.

RE: Variations in sample sizes across analyses are not clear

We would like to clarify the reason for our varying sample sizes: Our analysis is split into two sections. In the first section we grouped players based on their practice schedules using a rule-based approach inspired by previous research. These groups, which we view as the observational analogue to experimental conditions, were extracted from our pre-processed sample of 162k players. Recognising a limitation in our rule-based subsampling, we conducted a novel exploratory analysis in the second section, this time grouping different practice schedules using a data-driven, machine learning approach. As we wished to compare this second, alternative method with our first approach, we performed our clustering analysis on the pre-processed sample of 162k. We have added some text to the first paragraph of 3.1 to make this clearer.

In both sections we are selecting groups of players that meet our "experimental criteria" (e.g.., massing versus spacing practice), which relates to play behaviour that is atypical of the larger population. Subsampling players engaging in atypical behaviour naturally reduces our group sizes. Further, having recognised that the correlation between initial ability and play intensity confounds our analysis, in both sections of our analysis engaged in additional filtering, subsampling players on initial ability (based on a range surrounding the median performance) prior to statistical analysis. This resulted in further reduction to our group sizes.

In sum, as our research question relates to particular groups of players who behave in specific ways, we argue that it is not unusual for the sizes of groups in our analysis to be a small fraction of the entire sample. Instead, they are the result of a series of theoretically motivated filtering steps. We have taken steps to ensure that these filtering steps are clearly described in paragraphs 2 and 3 of the Results section (see also rebuttal point below on subsampling players by day range).

The example R2 provides of a group numbering less than 400 is a result of these same filtering steps: 1) following preprocessing we retained ~162k players, 2) matching on initial KDA we retained ~17k players, 3) of these players, ~400 played took between 136-150 days to play their first 100 ranked matches. Other groups in our analysis contained more players than this particular group. This relates to the highly skewed distribution of time gap between first and 95th match (see S2 Fig below).

It is worth noting that this group of ~400 players and the other groups in our statistical analyses are not related to the groups displayed in Table 3. This table serves only to describe the number of players that were shared between each pair of groups arising from the first (rule-based clustering) and second (ML clustering) section of our analysis. We did not conduct any statistical analyses using groups of sizes displayed in Table 3, it is a descriptive table of joint frequencies only. To make this clearer, we have extended the caption for Table 3. We trust that these steps coupled with the explanations provided above clarify the discrepancies relating to our sample sizes.

RE: The three ranges of days used to subsample players should be clearly motivated in text

The day ranges used to subsample players were motivated by visualising the frequency distribution of the time elapsed in days between the first and last match for each player (S2 Fig, below), and choosing three groups that were sufficiently discrete from one another and that contained adequate sample sizes for analysis. We have addressed R2's concern by including this previously absent description in the Results section and referencing the figure.

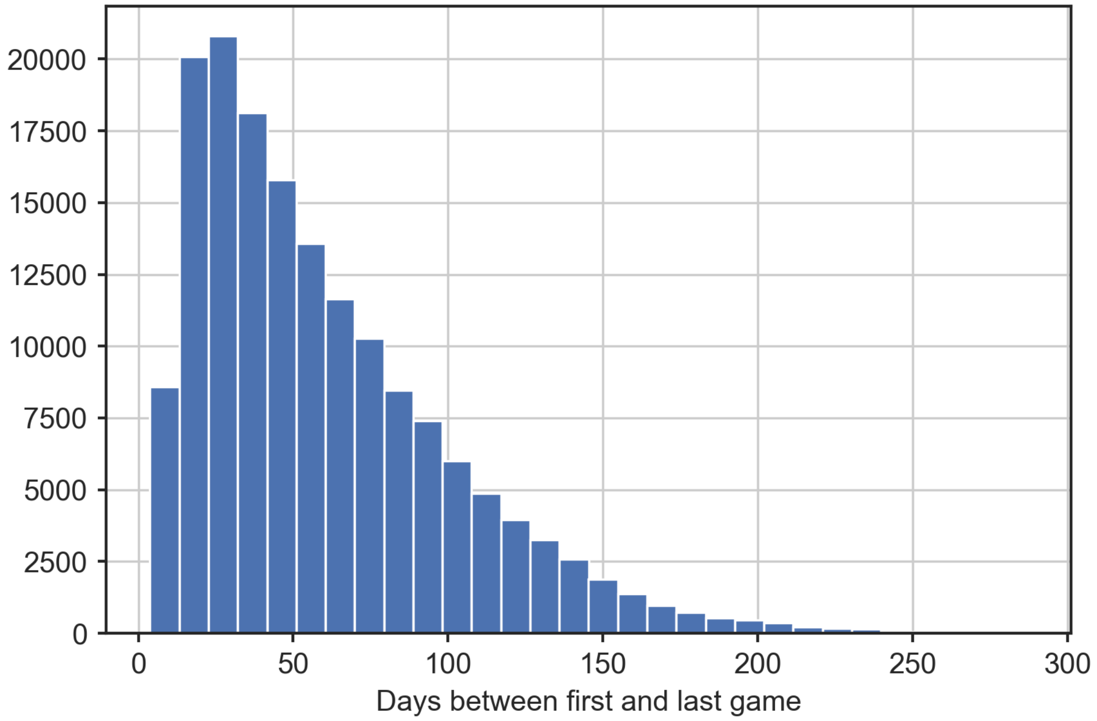

RE: Figure 3 displays time gap in hours, but text refers to time gap in days

The x axis in Figure 3 should indeed be labelled as time gap in days. We have corrected the x axis label and made sure the x axis metric is clearly described in the figure caption.

Plots depicting our machine learning clusters refer to time gap in hours, not days, as players were clustered based on their time series of inter-match time gap We have checked to ensure that this is clearly described in the section describe our clustering, and included a description of the time gap metric in every relevant figure caption for our clustering analysis (Figure 4, Figure 5, S5 Fig, S6 Fig).

RE: Discussion around figures relating to clustering analyses could be improved

We thank R2 for raising important points surrounding the discussion of our clustering analysis in section 3.1. Our discussion of how matches were temporally distributed does indeed center on figure 5, not 6. A further mistake involves our describing cluster 4 as the group of players with the earliest and shortest rest period, when the text should actually refer to cluster 3 as suggested. We have corrected these two mistakes, and also restructured the remainder of the paragraphs describing our clustering analysis to make the section clearer overall.

RE: Suggestions regarding improvements to figures

We agree with R2 that density is difficult to discern the density of points in Figure 3. We suggest that a scatter plot may be better at displaying the relationship between our two variables than the proposed heat map, but have elected as a middle-ground option to reduce the size and opacity of our points in our scatterplot to make density more apparent.
