# Supplementary material for: Mind the gap: Distributed practice enhances performance in a MOBA game
Source: PLoS One. 2022 Oct 14;17(10):e0275843. doi: 10.1371/journal.pone.0275843 (PMC9565695; doi:10.1371/journal.pone.0275843)
Supplement: Attachment — Submitted filename: PONE-D-22-03288_Second_Rebuttal_Letter.pdf [file pone.0275843.s012.pdf]

PONE-D-22-03288 Second Revision

Ozan Vardal
Department of Computer Science, University of York

20th September 2022

Dr Musa
Attached is our second revision of "Mind the gap: Distributed practice enhances performance in a MOBA game" [PONE-D-22-03288]. We thank the editor and reviewers for their careful inspection of our previous rebuttal and for leaving their final comments. We have made several changes in light of your latest responses, which we outline below.
We look forward to further updates on our paper.
Kind regards,
Ozan Vardal, Valerio Bonometti, Anders Drachen, Alex Wade, Tom Stafford

RE: Please review your reference list [...]

In line with the specified journal requirements for mentioning any changes to our reference list, we would like to clarify the following changes to our references:

References to MacQueen et al. (1967), Tavenard et al. (2020), Campello et al. (2013), and McInnes et al. (2017) were remnants of a previous iteration where appendix methods existed in the main body text. These citations no longer exist in the main body manuscript and have therefore been removed from the reference list.

In response to initial reviews, we also made significant changes to our concluding paragraphs. One paragraph including references to Baddeley and Longman (1978) and Schmidt and Lee (2019) was removed, resulting in removal of these papers from our reference list. In reframing our final paragraph, we included a supporting reference to Kokkinakis et al. (2017), which introduces readers to relevant work relating to potentially confounding variables that we mention in our discussion, and that are in our domain of interest.

RE: When replying to my comment about the MMR, the authors also notice that "Although [MMR] is somewhat dependent on match outcome, match outcome itself is dependent on many factors including the behaviour of teammates and opponents." I think the authors should actually add this observation to the discussion about MMR in the paper.

We agree with Reviewer 2 that this is helpful clarification for the reader. We have expanded our paragraph in the Discussion section on MMR to state:

Moreover, although MMR is partly dependent on match outcome, the probability of winning a match is dependent on many factors (including the behaviour of teammates and opponents), and is itself the subject of many efforts in prediction.

RE: In the edited text about ranked/unranked matches the authors write "our results 2". Should this be Figure 2?

We thank Reviewer 2 for pointing out this confusing sentence, which is indeed intended to reference Figure 2. We have amended it to "[...] than *what is observed in Figure 2* [...]".
